# Meibomian Gland Dysfunction Correlates to the Tear Film Instability and Ocular Discomfort in Patients with Pterygium

**DOI:** 10.1038/srep45115

**Published:** 2017-03-24

**Authors:** Huping Wu, Zhirong Lin, Fan Yang, Xie Fang, Nuo Dong, Shunrong Luo, Xumin Shang, Wei Li, Zuguo Liu

**Affiliations:** 1 Eye Institute and Affiliated Xiamen Eye Center of Xiamen University, Xiamen, Fujian, 361001, China; 2 Fujian Provincial Key Laboratory of Ophthalmology and Visual Science, Xiamen, Fujian, 361005, China

## Abstract

Pterygium is a very common disease in an eye clinic characterized by a benign proliferation of local conjunctiva that often crosses the limber of cornea and extends into corneal surface. Variety of studies has showed that pterygium is able to result in ocular discomfort and the change of ocular surface environment, such as dry eye. However, the link between abnormal tear film function and pterygium is controversial. Meibomian gland dysfunction (MGD) is a common cause of dry eye and ocular discomfort but is often neglected, which may be the missing link between dry eye and pterygium. In this study, our data firstly revealed increased abnormality of meibomian gland structure and function in pterygium patients, representing with increased abnormality of MGD parameters such as meibum expression (*P* < 0.001) and meibomian gland loss (*P* < 0.001). Besides, the scores of MGD severity in patients with progressive pterygium were higher than those in patients with resting pterygium. The correlation between MGD parameters and ocular discomfort as well as dry eye indexes is also established. These findings suggest that MGD correlates to the tear film instability and ocular discomfort in patients with pterygium.

Pterygium is a common disease in an eye clinic characterized by a benign proliferation of local conjunctiva that often crosses the limber of cornea and extends into corneal surface. It is named pterygium for the similarity of shape as the wing of insect^1^. The studies have showed that pterygium is able to result in the change of ocular surface environment, such as dry eye, which also is a risk factor for development of pterygium^1^,^2^.

Despite that there are many studies on the pterygium and itself function of tear film^3^,^4^,^5^,^6^, the correlation between the two is controversial. Some researchers reported that the epithelial cells of pterygium are in a status of high proliferation and differentiation, which resulted in a disability of mucin secretion or abnormal mucins leading to the abnormal protein layer of tear film^7^,^8^. Thereby the integration of tear film was greatly affected resulting in dry eye. Chan *et al*.^8^ found that the conjunctiva in patients with pterygium had squamous metaplasia and reduced density of goblet cells. Mustafa Ozsutcu *et al*.^2^ observed that the tear osmolarity and fluorescein sodium staining score were significantly higher while the parameters of BUT and Schirmer I test were significantly lower in 65 eyes with pterygium than those in contralateral control eye. Furthermore, other scholar reported that pterygium itself could lead to a local conjunctival elevation and an uneven distribution of tear resulting in dry eye with tear dynamics abnormality^7^. However, the relation between pterygium and tear film remains unclear and requires further investigation.

Meibomian gland (MG) function has been recognized as a critical factor in maintaining the ocular surface health and stability^9^. MG is a tubuloacinar sebaceous glandula that perpendicularly lied within tarsal. Each gland consists of central duct and gland alveoli and has opening at gray line of palpebral margin. The function of MG is to synthesize and secret lipids, which distributes to the ocular surface becoming the outmost layer of tear film and keeping the stability and reducing the evaporation of tear film^10^. Meibomian gland dysfunction (MGD) is a joint name that caused by all chronic and diffuse MG abnormalities characterized by a MG terminal blockage and/or the abnormality of materials of secretion/quantity^11^. The epidemiology^12^ reveals an very high incidence and the Asia has a higher percentage at 49.2–69.3%. Clinically, MGD is a common cause of evaporative dry eye and ocular discomfort but is often neglected. Our study has found that it is well related that the degree of dry eye depends on the intensity of discomfort and symptoms in patients with terminal of video-frequency syndrome^13^. Furthermore, it is observed in clinical work that pterygium patients are often accompanied by a MGD. Thus the attention of the relationship between MGD and dry eye was caught by our group. Currently, no study has been performed or reported on this point. We herein attempted to demonstrate and analyze the relationship between the meibomian gland function and parameters of dry eye as well as ocular discomfort.

## Results

### Features of Ocular Surface Disorders in Patients with Pterygium

The demography and parameters of dry eye in both groups were shown in Table 1. No significant differences of the age and sex ratio were found between the two groups (*P* > 0.05). Most patients with pterygium complained of mild to severe ocular discomfort. The OSDI values were more than 13 in 96.6% (57/59) of the pterygium patients. The OSDI value was significantly higher in pterygium patients than that of volunteers (20.05 ± 8.09 and 12.00 ± 5.16, respectively, *P* < 0.001), while the NIBUT was apparently lower than that of volunteers (5.39 ± 2.72 and 8.29 ± 3.96, respectively, *P* < 0.001). However, no differences between the pterygium group and the controls were found in tear volume (11.41 ± 6.21 mm and 13.23 ± 6.66 mm, respectively) either the corneal FL scores (0.43 ± 0.81 and 0.54 ± 0.90, respectively) (*P* > 0.05). Interestingly, the tear volume of the both group was in the normal range (above 10 mm).

### Abnormality of Meibomian Gland in Pterygium Patients

**Figure 1:**
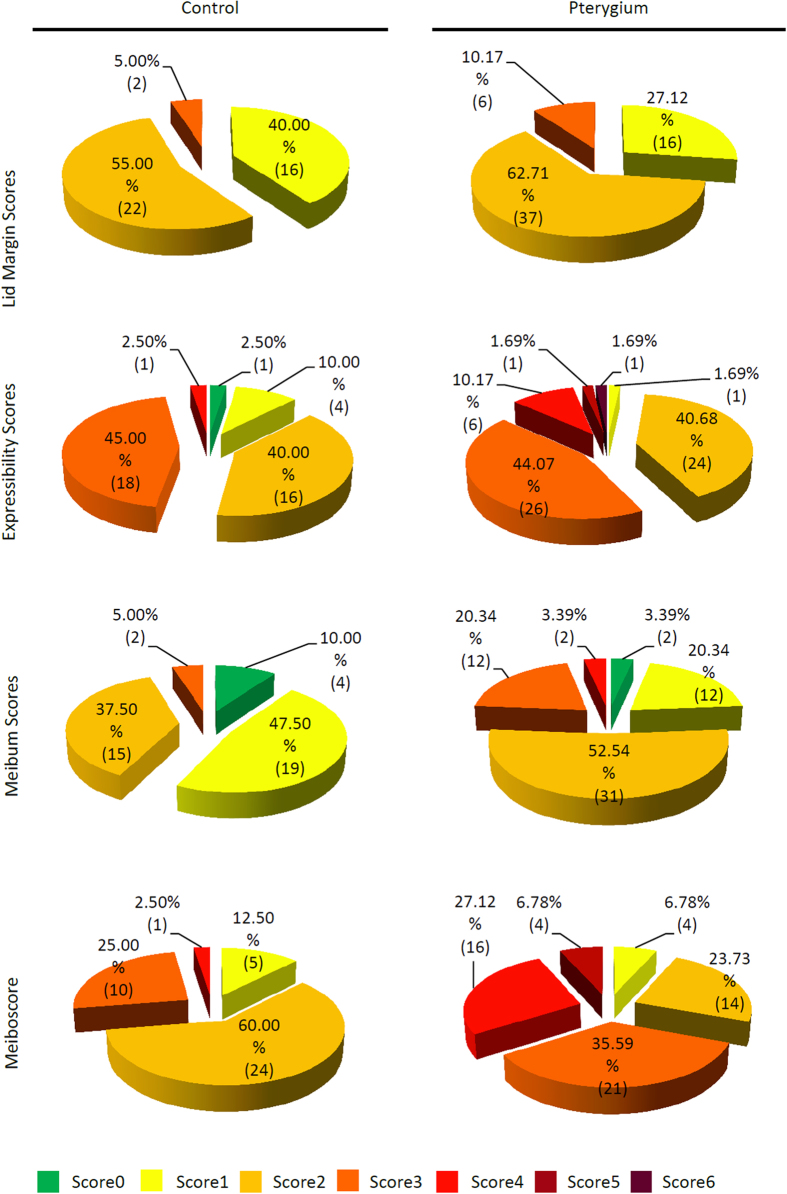
The detailed distributions of each meibomian gland parameters in the two groups. (n = 40 in the control group, n = 59 in the pterygium group).

The parameters of meibomian gland in both groups were shown in Table 2. The expressibility score, meibum score as well as meiboscore were significantly higher in the pterygium group than those of the controls (*P* < 0.05). However, No difference of lid margin scores between the two groups was recorded (*P* > 0.05). The detailed distributions of each meibomian gland parameters in the two groups were shown in Fig. 1. Abnormal meibum expression (meibum score ≥ 3) was observed in 14 eyes (23.7%) in the pterygium group while only 2 eyes (5%) in the control group. Apparent meibomian gland loss (meiboscore ≥ 3) was seen in 41 eyes (69.5%) and 11 eyes (27.5%) in these two groups respectively.

### Meibomian Gland Dysfunction in Patients with Progressive or Resting Pterygium

**Figure 2:**
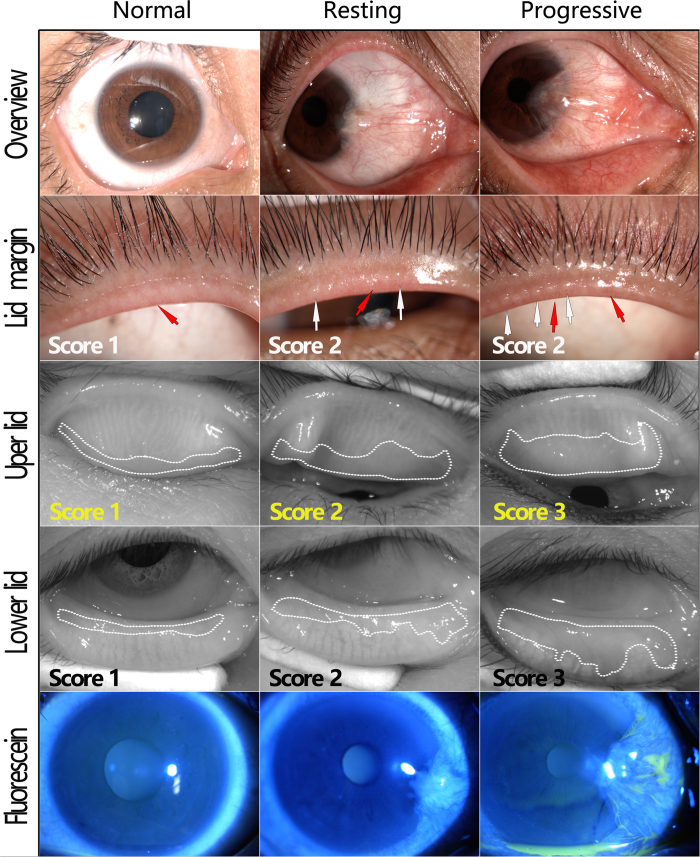
Representative images of lid margin, meiboghraphy of eye lids and fluorescein staining (Fluorescein) in patients from resting or progressive pterygium groups and normal controls. For lid margin and meibography of the eye lids, the score of each image is noted at bottom left. The left column shows normal eyes: the right eye from a 55-year-old female, OSDI: 13; NIBUT: 10.36 s; Schirmer test: 14 mm; the expressibility score was 2, and the meibum score was 1. The middle column shows eyes from resting pterygium group: the right eye from a 54-year-old female, OSDI: 19; NIBUT: 6.48 s; Schirmer test: 13 mm; the expressibility score was 2, and the meibum score was 2. The right column shows eyes from progressive pterygium group: the right eye from a 56-year-old female, OSDI: 26; NIBUT: 4.12 s; Schirmer test: 11 mm; the expressibility score was 3, and the meibum score was 3. The vascular engorgement is outlined by red arrow and plugged meibomian gland orifice is outlined by white arrow. The area of meibomian gland dropout was encircled with dotted white lines. Very few punctate corneal fluorescein staining in noted in pterygium eyes.

To determine whether the disease stage of pterygium has impact on meibomian gland function, the 59 eyes of the pterygium group were divided into two subgroups, the “resting stage” group (27 eyes) and the “progressive stage” group (32 eyes). The parameters of meibomian gland in these groups were shown in Table 3. The MG expressibility score of the resting stage group was higher than that of normal controls (1.83 ± 0.81 and 1.38 ± 0.74, respectively, *P* < 0.001), but no differences were found in the other three MG parameters between these two groups (*P* > 0.05). The expressibility score, meibum score as well as the meiboscore were apparently higher in the progressive stage group than those of normal controls (*P* < 0.05). Besides, the meibum score and meiboscore in the progressive stage group were much higher than those of the resting stage group (*P* < 0.001). The representative images of pterygium and meibomian gland parameters as well as corneal fluorescein staining from typical patients of each group were shown in Fig. 2.

### Meibomian Gland Dysfunction in Pterygium Patients with Long or Short NIBUT

Table 1 had shown that the patients with pterygium presented higher OSDI value and lower NIBUT, indicating the disrupted tear film stability. But meanwhile, most of these patients with dry eye condition had normal tear production. Accordingly, Table 2 revealed the increased abnormality of meibomian gland in pterygium patients. Based on these data, we speculated the functional status of meibomian gland correlated to the tear film stability and ocular discomfort in patients with pterygium. Thus we further divided the patients with pterygium into two subgroups according to NIBUT value: the short BUT group (NIBUT ≤ 5 s, n = 36) and long BUT group (NIBUT > 5 s, n = 23). The parameters of dry eye and meibomian gland function in the both subgroups were shown in Table 4. No statistic differences in tear volume, corneal FL scores, lid margin scores as well as the MG expressibility scores were found between these two subgroups (*P* > 0.05). However, the OSDI value, meibum scores and meiboscores were much higher in the short BUT group than those of long BUT group (*P* < 0.05), suggesting that the tear film instability and ocular discomfort might be associated with the abnormal lipid expression and meibomian gland loss.

### Correlations of Pterygium Parameters with Dry Eye Index and Meibomian Gland Function

The size, hyperemia and transparency are three of the major parameters in the clinical evaluation of pterygium. As shown in Table 5, in patients with pterygium, Spearman correlation analysis revealed that the hyperemia index of pterygium is significantly correlated with dry eye symptoms OSDI, meibum score as well as meiboscore (*P* < 0.05), while the size index of pterygium is significantly correlated with meiboscore (R = 0.325, *P* < 0.05). The transparency index of pterygium is also significantly correlated with meiboscore (R = 0.368, *P* < 0.01), but inversely correlated with NIBUT (R = −0.180, *P* < 0.05).

### Correlation of Meibomian Gland Dysfunction Parameters with Dry Eye Index

As shown in Table 6, Spearman correlation analysis revealed that the meibum score is significantly correlated with OSDI value (R = 0.208, *P* < 0.05), while inversely correlated with NIBUT (R = −0.307, *P* < 0.01). The meiboscore is also significantly correlated with OSDI value (R = 0.299, P < 0.01), but inversely correlated with NIBUT (R = −0.425, *P* < 0.001).

## Discussion

It was generally considered that tear film instability in pterygium patients may arise from two major factors: altered tear dynamics and chronic ocular surface inflammation. In the past decades, the detailed relationship between pterygium and tear film function has been extensively investigated^2^,^3^, however, no consistent standpoint was gained. On the other hand, the role of MGD in the tear film instability in various ocular surface diseases such as pterygium is not fully understood yet. In this study, the increased severity of dry eye and abnormality of meibomian gland in pterygium patients were revealed, representing with higher meibum score and meiboscore. The abnormality of meibomian gland was found to be more severe in patients with progressive pterygium than those with resting pterygium. Besides, most parameters of MGD were significantly correlated with dry eye index in pterygium patients. These results suggested that MGD plays an important role and is correlated to the discomfort and tear film instability in patients with pterygium.

One of our major findings is the increased abnormality of meibomian glands in pterygium patients. MGD is a chronic and diffuse disorder occurs in meibomian glands^11^. The etiology of MGD includes primary causes which are not fully understood, and secondary causes including ocular disorders such as blepharitis, conjunctivitis, etc., and systemic disease such as lupus erythematosus, Sjogren syndrome, etc^12^. Previous studies have discovered that meibomian gland function can be affected by various factors, such as overuse of video terminals^13^, etc. However, no study has focused on the role of pterygium in MGD so far. Our data showed that abnormal meibum expression (meibum score ≥ 3) was observed in 23.7% of the pterygium patients while only 5% in the control group. Apparent meibomian gland loss (meiboscore ≥ 3) was seen in 69.5% of the pterygium patients and only 27.5% in the controls. Higher scores of MGD parameters were also revealed in patients with progressive pterygium when compared with those with resting pterygium. Besides, the Spearman correlation analysis also revealed that the pterygium parameters were correlated with some of the MGD parameters (Table 5), indicating that the development of pterygium may be associated with MGD.

To get further understand on the importance of MGD in dry eye condition in pterygium patients, we divided the pterygium patients into two subgroups according to the value of NIBUT. The OSDI value, meibum score and meiboscore were significantly higher in the short BUT group than those of long BUT group (*P* < 0.05). These data suggested that the tear film instability and ocular discomfort might be associated with the abnormal functional status of meibomian glands. The Spearman correlation analysis also revealed that the meibum score and meiboscore were inversely correlated with NIBUT (Table 6). Taken together, MGD may probably play a critical role in the tear film instability in patients with pterygium.

Most of the past research focused on tear function in pterygium patients but drew different conclusions^14^,^15^,^16^. Our study supported that the tear film stability in pterygium patients could be significantly reduced while the tear volume remained normal, and MGD might contribute to the tear film instability. However, it was difficult to clarify the chronological sequence of the development of pterygium, MGD and dry eye. Based on our data, we hypothesized that the existence/development of pterygium might lead to tear dynamics alteration and MGD simultaneously, and the latter aggravated the tear film instability in turn. In fact, the hypertrophic and hyperemic pterygium may directly contact with the palpebral conjunctiva and even compress towards the beneath meibomian glands for years. Besides, the inflammatory factors associated with pterygium might also infiltrate and disrupt the microenvironment of MG thus leading to the MGD. However, the detail of the cause-effect relationship among them and the molecular mechanisms needs further investigation.

In conclusion, our data revealed increased abnormality of meibomian gland structure and function, and the correlation between MGD and ocular discomfort and tear film instability in pterygium patients. MGD may play a critical role in the development of tear film instability. These findings also suggest that clinical doctors need to pay more attention to the status of meibomian gland function in pterygium patients.

## Methods

### Patients

This study was approved by the ethics committee of Xiamen Eye Center, an affiliate of Xiamen University. All methods below were performed in accordance with the relevant guidelines and regulations. Written informed consent was obtained from participants in this study in accordance with the Declaration of Helsinki and its subsequent revision. Informed consent for online open-access publication of images or information from participants was also obtained.

This is a prospective, single center, randomized, controlled study. 59 patients with pterygium (pterygium group, 59 eyes) and 40 health volunteers (control group) were enrolled in the study at Xiamen Eye Center, an affiliated hospital of Xiamen University from Oct. 2014 to Dec. 2014. The pterygium group consisted of 10 males and 49 females, ranging in age from 32–72 years old, average (54.31 ± 8.58) years old. Volunteer group defined as control group consisted of 12 males and 28 females, age ranged from 31–74 years old, average (52.48 ± 11.35) years old.

The criteria for enrollment included patients with idiopathic pterygium in one single eye. The criteria for exclusion included those who had cataract surgery, allergic conjunctivitis, infectious corneal/conjunctivitis disease, contact lens wearing, topical application of artificial tears, ocular trauma or surgery, and any systemic diseases that affected the synthesis and function of tear or lipid.

All subjects were observed and examined by the followings: Ocular Surface Disease Index (OSDI), non-invasive tear film break up time (NIBUT), corneal fluorescein staining (FL) and Schirmer I test. The criteria of evaluation were mainly in accordance with MGD Workshop Report^17^ (2011) with slight modification as follows. The lid marginal abnormality, meibomian gland expressibility and the property of secretion liquid were examined and graded by a slit lamp. The characteristics MG dropout were evaluated by a Keratograph 5M (OCULUS, Germany). Pictures of each patient were taken by a slit lamp cameras and objectively graded for size, hyperemia, and transparency of pterygium. All procedures were performed by the same ophthalmologist in a dark room.

### Evaluation of Ocular Surface Disease Index (OSDI)

The symptom and intensity of dry eye were evaluated by a questionnaire, which is currently available and reliable. 12 questions were included and the answers according to symptoms in recent one week from patients were graded ranging from 0 to 100.

### Detection of non-invasive tear film break-up time (NIBUT)

NIBUT was analyzed by a Keratograph 5M (OCULUS, German). Briefly, Patient was sitting in the front of Keratograph 5 M laying the lower jaw on a mandible support. According to the manufactory’s instruction, a placido of 22 red concentric circles was directly projected to the surface of patient who was instructed to blink eye for twice keeping eye continuous open and gaze at central red spot. The NIBUT was calculated during continuous eye open by the device. The picture of the point by point and size of tear break of tear film was clearly displayed on the screen.

### Examination of corneal fluorescein staining (FL)

Four quadrants of corneal surface staining were examined using cobalt blue by a slit lamp (BQ900IM9900, Haag-Streit, Switzerland) 90 minutes after a corneal surface of patient was stained by a fluorescent paper. Cornea was divided into nasal superior, nasal inferior, temporal superior, and temporal inferior and graded as follows: 0: no staining, 1: slight scatted staining, 2: moderate staining between 1 and 3, 3: severe staining. Each quadrant was graded and the total score were 12 points.

### Schirmer I test

After the completion of the above three procedures and 30 minutes break, patients were tested with Schirmer I test paper placing in a 1/3 of middle to lateral conjunctival sac without topical anesthesia. Patients were asked to close eye for 5 minutes and then the paper was withdrawn from the eye.

### Observation of Lid Margin

Under the diffusing light of slit lamp, the abnormality of eyelid margin was evaluated^18^. The abnormality of eyelid margin (Lid Margin Score) was defined by the following four factors: lid margin irregular, score 1; vascular engorgement, score 2; glandular orifices obstruction, score 3; and anterior or posterior displacement of the mucocutaneous junction, score 4. No presence of above 4 is indicated as score 0.

### Expressibility and Lipid Secretion of MG

Under the diffusing light of slit lamp, central five meibomian glands were observed and squeezed. The activity of MG (expressibility score) was graded^17^ as follows: secretion was seen in all 5 meibomian glands, score 0; 3–4 glands, score1; 1–2 glands, score 2; none of the 5 glands, score 3. The final expressibility score of one eye including both lower and upper eyelid was 0–6. The property of lipid secretion of MG (meibum score) is scored^19^ as follows: clear or slight yellow, score 0; creamy yellow, score 1; granular in liquid with white and/or yellow color, score 2; tooth paste shape, score 3. The total score of each eye was 0–6, including the upper and lower eyelid.

### Non-contact infrared meibography

Patients were sit in front of the equipment (Keratograph 5 M, OCULUS, Germany) and the low chin was rested on the chin. The meibomian gland dropout was assessed using Meibo-Scan Program according to the manufactory’s instruction. The meibomian gland loss (Meiboscore) was scored^18^ as follows: no absence, score 0; absence less than 1/3 of total glands, score 1; absence more than 1/3 but less than 2/3 of total glands, score 2; absence more than 2/3 of total glands, score 3. The final score of each eye was 0 to 6 points including the upper and lower eyelid.

### Assessment of Pterygium

The property of pterygium was assessed under slit lamp. The disease stages of pterygium include “resting stage” and “progressive stage”. For resting pterygium, the tissue presents thin and flat with mild or even no hyperemia, but will not regress spontaneously. For progressive pterygium, the tissue appears large and wide body, hypertrophy with hyperemia and abundant vessels. The beneath sclera could not be observed clearly. In severe cases, the head of progressive pterygium will invade into the central cornea and cover the pupillary zone.

Pterygium size was graded according to the advancing edge position^20^: grade 1, less than 1/4 of corneal diameter; grade 2, more than 1/4 but less than 1/2 of the corneal diameter; grade 3, more than 1/2 but less than 3/4 of the corneal diameter; grade 4, more than 3/4 of the corneal diameter but outside the pupil center; grade 5, more than the corneal diameter but within the pupillary area; grade 6, the advancing edge across the pupillary area.

Under the diffuse light of slit lamp, the hyperemia of pterygium was evaluated into 4 grades as follows. Thin and flat pterygium body without hyperemia, score 1; thin and pink pterygium body with mild hyperemia, score 2; hypertrophic and red pterygium body with moderate hyperemia, score 3; apparently hypertrophic and cardinal red pterygium body with severe hyperemia and buckling of vessels, score 4.

The transparence grade of pterygium was evaluated as follows. Grade 1, the beneath sclera and the superficial sclerotic vessels could be clearly observed through the pterygium tissue. Grade 3, the sclera and the sclerotic vessels could not be seen through the pterygium. Grade 2 referred to the condition between grade 1 and 3.

### Statistical Analysis

All statistical analyses were performed by using SPSS V.16.0 software (SPSS Inc.; Chicago, IL, USA). Measurement data was presented with mean and standard deviation. Enumeration data was analyzed using Chi-square test; difference among the two groups was performed by using the Mann-Whitney U test, while differences between groups were analyzed by using the One-way ANOVA. When estimating the correlations between various factors, Spearman correlation analysis was used. The differences were considered statistically significant when the value of *P* < 0.05.

## Additional Information

**How to cite this article:** Wu, H. *et al*. Meibomian Gland Dysfunction Correlates to the Tear Film Instability and Ocular Discomfort in Patients with Pterygium. *Sci. Rep.* **7**, 45115; doi: 10.1038/srep45115 (2017).

**Publisher's note:** Springer Nature remains neutral with regard to jurisdictional claims in published maps and institutional affiliations.

## Figures and Tables

**Table 1:** Demography and Dry Eye Condition in Patients with Pterygium and the Controls.

	Controls (n = 40)	Pterygium (n = 59)	*P*
Age	52.48 ± 11.35	54.31 ± 8.58	0.436
Sex Ratio (F/M)	28/12	49/10	0.125
OSDI	12.00 ± 5.16	20.05 ± 8.09	<0.001
Schirmer I Test (mm)	13.23 ± 6.66	11.41 ± 6.21	0.189
NIBUT (s)	8.29 ± 3.96	5.39 ± 2.72	<0.001
Corneal Fluorescein Staining	0.43 ± 0.81	0.54 ± 0.90	0.554

**Table 2:** Scoring of Meibomian Gland Parameters in the Pterygium and Control Groups.

	Control (n = 40)	Pterygium (n = 59)	*P*
Lid Margin Score	1.65 ± 0.58	1.83 ± 0.59	0.137
Expressibility Score	2.35 ± 0.80	2.75 ± 0.1919	0.041
Meibum Score	1.38 ± 0.74	2.00 ± 0.83	<0.001
Meiboscore	2.18 ± 0.68	3.03 ± 1.03	<0.001

**Table 3:** Parameters of Meibomian Gland in Normal Control and Patients with Resting or Progressive Pterygium.

	Normal control (1) (n = 40)	Resting Stage (2) (n = 27)	Progressive Stage (3) (n = 32)	*P*	*P*		
					2 and 1	3 and 1	3 and 2
Lid Margin Score	1.65 ± 0.58	1.78 ± 0.60	1.86 ± 0.59	0.293	0.392	0.122	0.619
Expressibility Score	2.35 ± 0.80	2.69 ± 0.89	2.83 ± 0.86	0.065	0.078	0.033	0.559
Meibum Sore	1.38 ± 0.74	1.83 ± 0.81	2.22 ± 0.84	0.001	<0.001	0.004	<0.001
Meiboscore	2.18 ± 0.68	2.39 ± 0.84	3.44 ± 0.94	<0.001	0.654	<0.001	<0.001

**Table 4:** The Parameters of Dry Eye and Meibomian Gland in the Two Subgroups of Pterygium Patients.

	Normal control (n = 40)	NIBUT ≤ 5 s (n = 36)	NIBUT > 5 s (n = 23)	*P*
NIBUT (s)	8.29 ± 3.96	3.45 ± 0.58	7.44 ± 3.26	<0.001
OSDI	12.00 ± 5.16	22.36 ± 6.52	17.74 ± 4.87	<0.01
Schirmer I (mm)	13.23 ± 6.66	11.33 ± 5.76	10.95 ± 6.39	0.189
Corneal FL	0.43 ± 0.81	0.73 ± 0.91	0.45 ± 0.98	0.325
Lid Margin Score	1.65 ± 0.58	1.86 ± 0.63	1.78 ± 0.58	0.632
Expressibility Score	2.35 ± 0.80	2.87 ± 1.01	2.79 ± 0.65	0.482
Meibum Score	1.38 ± 0.74	2.30 ± 0.81	1.76 ± 1.05	<0.001
Meiboscore	2.18 ± 0.68	3.01 ± 0.78	2.83 ± 1.12	<0.05

**Table 5:** Correlations of Pterygium Parameters with Dry Eye Indexes and Meibomian Gland Functions.

	Hyperemia		Size		Transparency	
	R	*P*	R	*P*	R	*P*
OSDI	0.260	0.047*	−0.005	0.971	0.152	0.252
Schirmer I Test	0.205	0.120	0.124	0.349	0.112	0.399
NIBUT	−0.229	0.081	0.042	0.752	−0.180	0.036*
Corneal FL	0.028	0.830	−0.112	0.399	−0.215	0.102
Lid Margin Score	0.087	0.510	0.065	0.627	0.097	0.467
Expressibility Score	0.058	0.661	−0.050	0.704	0.048	0.717
Meibum Score	0.247	0.014*	−0.125	0.346	0.070	0.598
Meiboscore	0.788	<0.001**	0.325	0.012*	0.368	0.004**

Note: R, correlation value by Spearman correlation analysis; *P,* significance level in Spearman correlation analysis. *P < 0.05; **P < 0.01.

**Table 6:** Correlation of Meibomian Gland Dysfunction Parameters with Dry Eye Index.

	Lid Margin Score		Expressibility Score		Meibum Score		Meiboscore	
	R	*P*	R	*P*	R	*P*	R	*P*
OSDI	0.158	0.118	0.137	0.176	0.208	0.039	0.299	0.003
Schirmer I	−0.101	0.320	<0.001	0.997	−0.083	0.413	0.042	0.676
NIBUT	−0.001	0.996	−0.156	0.122	−0.307	0.002	−0.425	<0.001
Corneal FL	0.161	0.112	0.056	0.581	0.101	0.320	0.091	0.369

Note: R, correlation value by Spearman correlation analysis; *P,* significance level in Spearman correlation analysis.
